# Effect of Maternal Diet and Milk Lipid Composition on the Infant Gut and Maternal Milk Microbiomes

**DOI:** 10.3390/nu12092539

**Published:** 2020-08-21

**Authors:** Michal Dayagi Babakobi, Leah Reshef, Shalev Gihaz, Bogdan Belgorodsky, Ayelet Fishman, Yoram Bujanover, Uri Gophna

**Affiliations:** 1 The Shmunis School of Biomedicine and Cancer Research, Faculty of Life Sciences, Tel Aviv University, Tel Aviv 6900001, Israel; leahfa@gmail.com (L.R.); urigo@tauex.tau.ac.il (U.G.); 2 Department of Biotechnology and Food Engineering, Technion- Israel Institute of Technology, Haifa 3200003, Israel; shalevgihaz@gmail.com (S.G.); afishman@technion.ac.il (A.F.); 3 School of Chemistry, Tel Aviv University, Tel Aviv 69000, Israel; belgorod@post.tau.ac.il; 4 Safra Children’s Hospital, Sheba Medical Center, Ramat Gan 52621, Israel; Yoram.Bujanover@sheba.health.gov.il

**Keywords:** breast milk, human milk microbiome, human milk fat composition, infant gut microbiome, maternal diet

## Abstract

Inter-subject variability in human milk microbiome is well known; however, its origins and possible relationship to the mother’s diet are still debated. We investigated associations between maternal nutrition, milk fatty acids composition and microbiomes in mother–infant dyads. Breast milk and infant fecal samples were collected across three time points (one week, one month and three months postpartum) from 22 mother–infant pairs. Food frequency questionnaires for the months of pregnancy and three months postpartum were collected. Milk fatty acids were analyzed by GC–MS and the microbiome in breast milk and infant feces was determined by 16S rRNA sequencing. Statistical interactions were computed using Spearman’s method and corrected for multiple comparisons. We found significant negative correlation between *Streptococcus* relative abundance in maternal milk and intake of unsaturated fatty acids and folic acid at one month postpartum. At three months postpartum, vitamin B-12 consumption was significantly associated with a single operational taxonomic unit belonging to *Streptococcus*. Comparison between milk microbiome and lipid composition showed, one-month postpartum, significant negative correlation between *Streptococcus* relative abundance and the abundance of oleic acid. Additional correlations were detected between *Staphylococcus hominis* and two medium-chain saturated fatty acids. Our results reinforce the hypothesis that maternal nutrition may affect milk microbiome.

## 1. Introduction

Health organizations worldwide have determined that human milk is the most important source of nutrition for newborns in the first weeks and months of their lives. Today it is well-established that human milk is not only an important nutritional source for the newborn, but also provides fertile ground for the establishment and growth of her or his gut microbiota, during their early and critical period of life [1,2,3,4,5]. Recent studies have established a link between development of early-life gut microbiome, and its influences on short and long-term infant health outcomes [6,7]. The initial development of intestinal microbiome is important in establishing both intestinal and systemic immunity. Immediate consequences of infant gut microbiome dysbiosis can include growth impairment [8,9] and an increased risk of sepsis and necrotizing enterocolitis (NEC), especially in preterm newborns [10,11,12]. The associated long-term health consequences include allergies, metabolic syndrome, diabetes and inflammatory bowel diseases [3,13,14].

A study that examined the total bacterial numbers in breast milk showed high variability among different women and even at different time points in samples from the same woman. The median value reported was 10^6^ bacterial cells per ml, with no significant differences between colostrum, transition and mature milk [15]. Over 207 bacterial genera have been identified in human milk [16], the most prevalent being *Streptococcus* and *Staphylococcus* [17]. The human milk bacteria could serve as a potential probiotic source that colonizes the infant’s gut, and passage of bacteria from maternal milk to the infants’ gut was indeed demonstrated [18,19]. The structure of the human milk bacterial communities varies greatly, both between subjects and across time [16,20].

Several factors have been found to affect human milk microbiome, including genetics, environmental and geographic factors, and mode of delivery, as well as the mother’s health status, weight and nutrition [18].

In the context of human milk and its effects on the microbiome, milk oligosaccharides are considered to be the most influential component, which promotes the growth of specific bacterial taxa, especially bifidobacteria [3]. An additional important and complex component of breast milk, whose composition varies from mother to mother, similar to the composition of the milk microbiome, is the fat. The fat content in the human milk, particularly the long-chain polyunsaturated fatty acids (LC-PUFAs), is significantly affected by the mother’s diet [21,22]. It is known that about 75% of the linoleic acid in breast milk comes directly from the mother’s diet and about 30% is derived from the mother body’s fat stores [23,24,25], and its composition changes during lactation [26]. Another major lipid component of human milk is medium-chain fatty acids (MCFAs), mostly 12:0, and 14:0. The concentrations of MCFAs have been shown to be influenced by the amount of carbohydrate and fat in the maternal diet, whereby high carbohydrate and low fat in the diet increase MCFAs synthesis [27].

Here we explore the effect of maternal nutrition on both milk lipid content and microbiome, across multiple time points. We also investigate potential interactions with the infant gut microbiome. To address these issues, 22 mother–infant pairs were followed up for three months postpartum, and maternal milk samples, infant stool samples, and food frequency questionnaires were collected and analyzed.

## 2. Materials and Methods

### 2.1. Participants and Sampling

Twenty-four healthy women, aged 25–40, were recruited from the maternity ward in Sheba Medical Center Hospital, Tel Hashomer, one to two days after delivery. Clinical characteristics of mother–infant pairs are provided in Table S1. Inclusion criteria were healthy women without any known background diseases, who gave birth vaginally, had a normal birth process with a healthy newborn born at term. All mothers declared they will practice exclusive breastfeeding for the neonate until at least, three months of age. All subjects gave their informed consent for inclusion before they participated in the study. The study was conducted in accordance with the Declaration of Helsinki, and the protocol was approved by the Ethics Committee of Sheba Medical Center Hospital, Tel Hashomer (1424-14 SMC).

The mothers were instructed to take milk samples and infant fecal samples at three different time points (first week postpartum, one month and three months postpartum). Every research volunteer received a sampling kit that included: three sterile 50 mL test tubes for breast milk samples, three for fecal samples, six disposable nitrile gloves and written instructions for proper collection and storage, for standardization. Twenty-two women completed the study. After the samples were collected, they were stored at −20 °C for later analysis. Overall, 62 milk samples and 60 stool samples were collected. Five milliliters of milk was collected at the first time point and 10 mL at the subsequent time points; the subjects were instructed to sample milk from mid-breastfeeding. Samples were vortexed for homogenization before subsequent analyses.

### 2.2. Dietary Assessment

At the end of three months, when all the samples had been collected, the mother filled, under the guidance of a dietician, a food frequency questionnaire (FFQ), on her nutritional consumption habits during the past year (the nine months of pregnancy and three months after delivery). The FFQ was developed and validated for the Israeli population by the International Center for Health and Nutrition, Department of Public Health, Ben-Gurion University of the Negev [28], and includes 126 food items. The consumption frequency for every food item is graded on a nine-point answer scale from “never or once per month” to “more than five times per day.” Quantification of food intake was performed using standard serving sizes and different portion sizes for selected dishes. The questionnaires were analyzed on the United States Department of Agriculture (USDA) database. To verify that the responses in the FFQ truly captured participant dietary habits, the participants were asked to fill out a 24 h dietary recall questionnaire a day before the samples were taken, at all three time points, which was compared to the FFQ.

### 2.3. Analysis of Fatty Acids in Human Milk

The human milk fatty acid composition was determined using gas chromatography–mass spectrometry (GC–MS) analysis after chemical acidic transesterification of the milk triglycerides into fatty acid methyl esters (FAMEs) (Figure S1). The procedure was as described in the work of Cruz-Hernandez et al. [29], with few modifications. Briefly, a 250 μL human milk sample was mixed with 1.3 mL hexane, 2 mL methanol, 2 mL methanol/HCl 3N and 300 μL heptadecanoic methyl ester (17:0) hexane solution as an internal standard (unlike the 11:0 fatty acid used in the original work). The reaction mixture was sealed with a magnetic stirrer and agitated at 100 °C for 1 h. Following the reaction, the vials were cooled down and the organic phase was extracted and collected for analysis. The FAMEs content was analyzed and quantified by Gas chromatography–mass spectrometry (GC–MS). GC–MS analyses were performed on an Agilent 6890/5977A GC–MS (Eldan Electronic Instruments Co. Ltd., Petach-Tikva, Israel) system equipped with Agilent 30 m × 0.25 mm i.d. HP-5MS column (5% Phenyl/Methylpolysiloxane, 0.25 μm film thickness). The carrier gas was helium (99.999%) at a constant flow rate of 1.2 mL/min. The GC conditions were as follows: injection volume 1 μL; injector temperature 250 °C with split ratio of 1:5; the initial oven temperature was 70 °C with hold for 2 min and then increased to 180 °C at a rate of 5 °C/min with hold for 5 min, which was followed by raising the temperature to 240 °C at a rate of 20 °C/min and hold for 9 min. Finally, the temperature was raised to 300 °C at a rate of 30 °C/min with hold for 6 min. MS was performed in the EI positive ion mode at 70 eV electron energy. Transfer line temperature and ion source temperature were maintained at 280 °C and 230 °C, respectively.

MS data were collected at a range of 50–450 and analyzed with Chemstation software (Agilent Technologies, Eldan Electronic Instruments Co. Ltd., Petach-Tikva, Israel Ver. F.01.01.2317). The FAMEs were identified by comparing the retention times of standards (Supelco 37-FAME standard). Quantification was based on commercial external standards (Sigma, Rehovot, Israel).

### 2.4. Microbiome Composition Analysis

#### 2.4.1. DNA Extraction

Total DNA was extracted using PowerSoil DNA extraction kit (MoBio, Carlsbad, C.A. USA). The extraction process was conducted according to the standard manufacturer’s protocol (https://mobio.com/media/wysiwyg/pdfs/protocols/12888.pdf) except that for milk samples, 400 uL were added directly to the bead tube and along all the procedure, the volume of the added reagents was corrected proportionally to the sample volume. The swabs with the fecal samples were inserted into the tube and soaked with the bead solution. Subsequent steps followed the protocol of the manufacturer. Extracted DNA was stored at −20 °C.

#### 2.4.2. Amplicon Sequencing

Polymerase chain reaction (PCR) amplification of the 16S rRNA gene was carried out in a PCR cabinet with universal prokaryotic primers containing 5-end common sequences as previously described [30] (CS1-341F 5′-ACACTGACGACATGGTTCTACANNNNCCTACGGGAGGCAGCAG and CS2-806R 5′-TACGGTAGCAGAGACTTGGTCTGGACTACHVGGGTWTCTAAT). Each reaction consisted of 12.5 μL Kappa2G Fast DNA Polymerase, 1.25 μL from each primer, 1 μL of extracted template DNA and 9 μL ultrapure water. Thermal cycler settings included a 3 min denaturation step at 95 deg followed by 29 cycles of 95 deg for 15 s, 53 deg for 15 s, and 72 deg for 15 s. A final elongation step at 72 deg for 1 min was then performed to complete the reaction. PCR products were stored at −20 °C until further use. The PCR fragments were analyzed using DNA gel electrophoresis and checked for correct size (~450 bp). Samples producing exceptionally strong bands were re-amplified after a 5-fold dilution. Samples were then shipped to the Chicago Sequencing Center at the University of Illinois, where barcodes were added in an additional 8 cycle PCR reaction, and products were purified, pooled and paired-end sequenced on an Illumina MiSeq platform.

#### 2.4.3. Sequence Analysis

Demultiplexing and adaptor removal were performed at the sequencing center. Paired reads were then merged with PEAR (https://cme.h-its.org/exelixis/web/software/pear/), with a minimum quality threshold of Q20 and minimal sequence length of 410. The QIIME package (http://qiime.org/) was used for primer removal, operational taxonomic unit (OTU) clustering at 99%, filtration of chimeric sequences, and taxonomic assignment using the UCLUST (https://drive5.com/usearch/manual/uclust_algo.html) algorithm against GreenGenes database, V 13.8. In total, 2,424,700 sequences were retained at the end of the pipeline with a mean of 17,444 high-quality sequences per sample. To avoid bias due to differences in sequence depth, samples were rarified to an even depth of 4200 sequences per sample. UniFrac distance matrices were computed using QIIME.

### 2.5. Statistical Analysis

Spearman’s correlation method was used to assess interaction between microbial taxa (at the family, genus, or OTU level), human milk fatty acids, and nutrients consumed. Analyses were performed using R 3.1.2 [31]. The *p*-values were then corrected for multiple comparisons using the false discovery rate (FDR) method [32]. Results with *p* values < 0.01 and *q* (FDR) values < 0.05 were considered significant.

## 3. Results

### 3.1. Multiple Core Genera Are Present in Both Human Milk and Infant Stool

Complete mother–infant profiles, consisting of a maternal milk sample, an infant stool sample, and dietary questionnaires were obtained across all three time points for 16 mother–infant pairs. Six additional mother–infant pairs supplied complete information for two of the three time points. Overall, milk profiles were obtained from 62 samples, and 16S rRNA based microbiome analysis was conducted on 122 samples.

**Figure 1:**
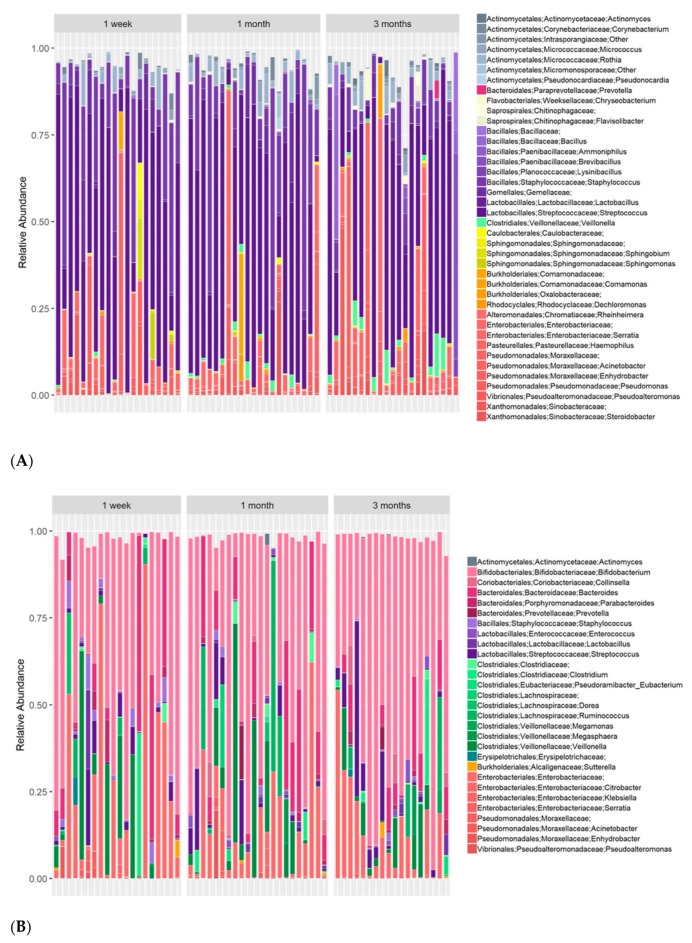
Per-subject longitudinal taxonomical profiles. (**A**) Maternal milk microbiome; (**B**) Fecal microbiome of corresponding infant. Genera whose abundance was <0.03% in all samples are not shown.

The maternal milk microbial composition highly differed from infant’s fecal bacterial composition (analysis of similarities (ANSOIM) *p*-value: 0.001, R = 82%; (Figure S2)), with milk samples enriched in oral, skin and environmental bacteria, and fecal samples dominated by typical gut obligatory anaerobes (Figure 1).

**Figure 2:**
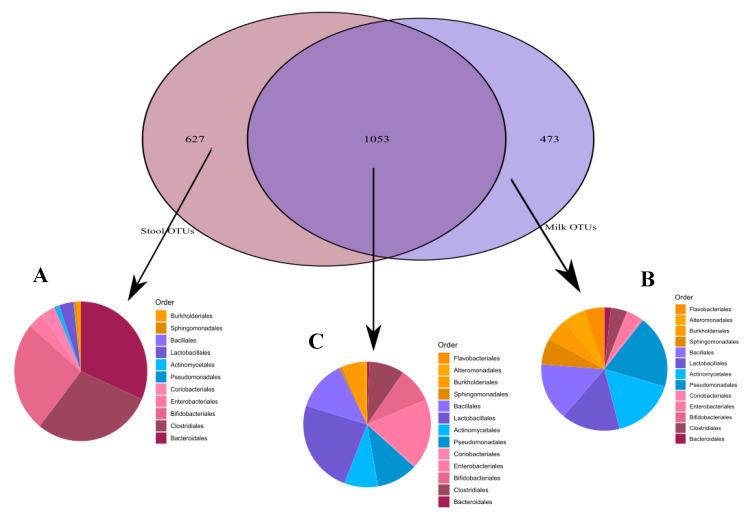
Operational taxonomic units (OTUs) order-level taxonomical assignments. (**A**) Stool-unique OTUs; (**B**) Milk-unique OTUs; (**C**) Shared OTUs (OTUs appearing in at least one milk and one stool sample).

However, 49% of the OTUs identified in this study were observed at least once in both niches. Notably, while most of the stool-specific OTUs could be assigned to strict gut anaerobe orders (*Bacteroidales*, *Clostridiales*, and *Bifidobacteriodales*), and most of the milk-specific OTUs assigned to orders that are considered more aerobic (*Bacillales*, *Lactobacillales*, *Actinomycetales*, and *Pseudomondales*), the shared OTU fraction contained members of all the above bacterial orders as shown in Figure 2.

A similar microbial core, defined here as genera appearing in over 90% of the samples, was found for both stool samples (*Bifidobacterium*, *Streptococcus*, *Escherichia* and *Staphylococcus*) and milk samples (*Streptococcus*, *Escherichia*, and *Staphylococcus*). Nevertheless, the relative abundance (RA) of core genera across the two sample types varied greatly, with Streptococcus and Staphylococcus being far more abundant in milk (mean RA 47%, and 15%, respectively) than in stool (mean RA 4.5% and 1%), and Escherichia being far more abundant in stool (mean RA 18%) than in milk (5%). Bifidobacterium, highly abundant in stools of the infants (mean RA 45%), was barely detectable in milk (mean RA 0.2%), presumably because it cannot replicate in oxygen-rich environments.

### 3.2. An Individual Signature in Both Milk and Infant Gut Microbiomes Is Retained across Time

**Figure 3:**
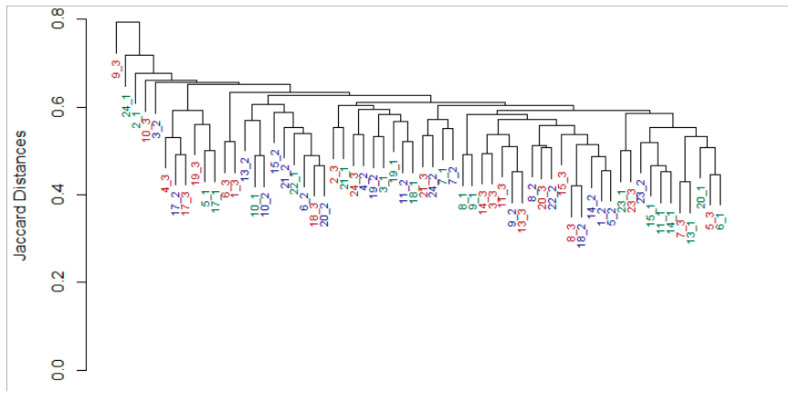
Subject identity in maternal microbiome. Dendrogram based on nearest neighbor joining of samples according to the Jaccard distance matrix of milk microbiome samples. Leaf labels show Subject ID and the time the sample was collected (green_week postpartum, red_one month postpartum, blue_three months postpartum). Analysis of similarities (ANOSIM): *p* = 0.002 R = 20%. No data were available for participants number 12 and 16.

To assess to what degree the milk microbiome is mother-specific and maintains temporal stability, we calculated pair-wise distances in microbial composition between every two milk samples using both abundance-weighted and unweighted community distance indexes. A weak but significant clustering of milk microbiome samples according to maternal identity could be observed when using the Jaccard presence/absence index (ANOSIM; *p*-0.002, R = 20%, see Figure 3).

**Figure 4:**
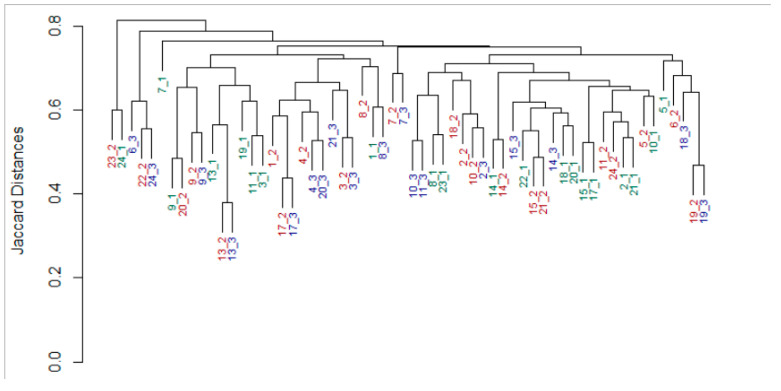
Subject identity in infant stool microbiome. Dendrogram based on nearest neighbor joining of samples according to the Jaccard distance matrix of stool microbiome samples. Leaf labels show Subject ID and the time the sample was collected (green_week postpartum, red_one month postpartum, blue_three months postpartum). ANOSIM: *p* = 0.001, R = 56%. No data were available for participants number 12 and 16.

A far stronger subject-specific signature was detected in the infants’ fecal microbiomes (*p* = 0.001, R = 56%, Figure 4). In addition, the variability between the gut microbiome of different infants (inter-subject) was high, and decreased with time, as observed in previous studies [33,34,35,36,37,38], (Figure S3).

### 3.3. Maternal Milk Microbiome Is Affected by the Mother’s Nutrition

Of the entire set of nutritional information obtained via the questionnaires, we selected 21 dietary parameters that varied considerably across subjects (Table S2) (had relative standard deviation > 40%). We then correlated microbial RA values in the maternal milk with those 21 parameters, for microbial taxa that had a median RA greater than 0.16%. Significant nutritional–microbial associations were obtained for the second time point, with *Streptococcus* being strongly and negatively correlated to the intake of unsaturated fatty acids and folic acid (Table 1). Since most bacterial genera in our data consisted of multiple species-level operational taxonomic units (OTUs, in this case defined as 16S rRNA gene sequences that had over 99% sequence identity), we repeated the correlation analysis using the relative abundance data of individual OTUs, rather than of bacterial genera, and obtained similar results (Table S3). Additionally, a significant association between vitamin B-12 consumption and a single OTU belonging to the *Streptococcus* genus was detected at the third time point (FDR < 0.001, R = −0.695, Table S4).

Analysis of the association between the mother’s dietary components, breast milk lipids, or breast milk microbiome, and the infant’s fecal microbiota, did not reveal statistically significant associations at any time point in this study.

### 3.4. Fatty Acid Composition in Maternal Milk Directly Affects Maternal Microbiome

**Figure 5:**
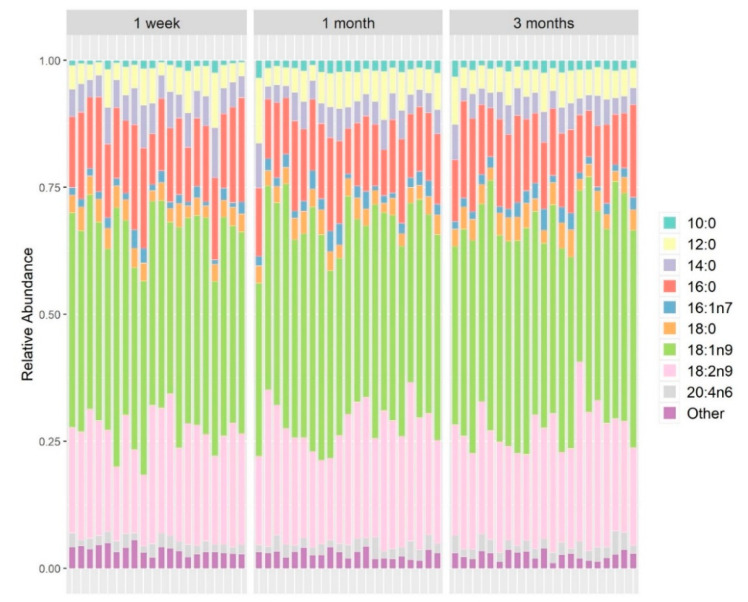
Fatty acids composition in the maternal milk samples. Fatty acids detected in quantities less than 2% (wt/wt) were grouped as “other”.

In the milk samples, we were able to identify 19 fatty acids, nine of which constituted the dominant part of human milk fat (Figure 5). In general, fatty acid composition did not differ substantially across mothers, and the composition remained relatively stable throughout the three months of the study. Saturated fatty acids constituted a lower fraction of milk lipids, as observed previously for Israeli women [39,40], and in contrast to other populations [27,41], probably as a result of the Israeli diet.

The concentrations of omega-3 fatty acids were not higher among women who took omega-3 supplements compared to those who did not take such supplements (0.56 ± 0.0041% vs. 0.54 ± 0.0043%, *p* = 0.83, Mann–Whitney U test).

All 19 fatty acids detected in the milk samples (Table S5) were used to test for associations with taxa from the milk microbiome at each of the three time points. A significant negative correlation was observed between *Streptococcus* RA and the abundance of the mono-unsaturated fatty acid, oleic acid (18:1(n-9)), the most abundant fatty acid in maternal milk (FDR < 0.001, R = −0.8), mirroring the correlation we previously observed between *Streptococcus* and dietary intake of monounsaturated fatty acids (see above). Again, the OTU-based analysis showed similar results to those obtained at the genus level. Nevertheless, the increased resolution of an OTU-based analysis did uncover an additional correlation, between two OTUs representing *Staphylococcus* hominis-related bacteria (at 99% 16S rRNA sequence identity) and two medium-chain saturated fatty acids in the milk, octanoic acid (8:0) (FDR < 0.001, *p* < 0.001, R = 0.7) and lauric acid (12:0) (FDR = 0.04, *p* = 0.001, R = 0.7). When we compered the composition of fatty acids in the mother’s milk to the mother’s nutrient components we saw a significant direct association, at the second time point, between the abundance of oleic acid in the human milk to the amount of monounsaturated fatty acids that the mother consumed in her diet (*p* = 0.006, R = 0.58). However, after correcting for multiple comparisons, those results did not reach statistical significance (FDR = 0.6). This trend nevertheless supports the other associations that we have observed and suggests that the effect of maternal nutrition on milk bacterial composition may be mediated, at least partly, by the fatty acids in her breast milk.

## 4. Discussion

The source, or sources, of bacteria in human milk remain controversial: bacteria can reach the mammary glands from external sources through retrograde flow, from internal source via entero-mammary pathway, or from a combination of the two sources [42,43]. Regardless of the relative contribution of these various sources, it has become generally accepted that bacteria in milk are not secondary contamination, but a distinct microbiome that differs from other microbiomes in the mother and infant [20,44]. Here, we explored temporal microbial patterns in maternal milk and corresponding infant stool, and their associations with milk lipid profiles and maternal nutrition. The infants in this study were exclusively breastfed. Distinct microbial profiles were found in milk and in stool; however, nearly half the OTUs identified in this study were detectable in both niches. In particular, typical beneficial gut anaerobes such as *Ruminococcus*, *Bacteroides*, and *Bifidobacterium*, the latter shown to be pivotal in development of the infant gut microbiome [45,46,47,48,49,50], were detected, at extremely low abundances, in milk samples. This observation supports the current view that maternal milk provides a microbial source for infant intestinal colonization [44,51,52,53]. Yet, the main effect of breast milk on the composition of the baby’s gut, does not necessarily come from the milk bacteria themselves, but rather from other milk components that enrich for specific bacterial groups, such as lactic acid bacteria. This could explain the lack of strong correlations between the bacterial composition of breast milk and the fecal microbiota composition of the infants. Overall, the strongest association we found was the negative correlation between the most common bacterial genus in the milk samples, *Streptococcus*, and the mother’s consumption of unsaturated fats. This relationship may be explained by the fact that fatty acids, especially unsaturated fatty acids, have antibacterial effects on certain bacteria, including *Streptococci*, for which direct inhibition has been documented [54]. A similar effect of monounsaturated fatty acids (a subgroup of the unsaturated fatty acids) dietary intake on the relative abundance of the genus *Corynebacterium* in maternal milk has been shown by Williams et al. [55]. We have observed a similar trend for *Corynebacterium* relative abundance that was negatively associated with consumption of unsaturated fats in our data as well, R = −0.67, *p* = 0.001, though that trend did not reach statistical significance after correction for multiple hypothesis testing (FDR Q = 0.273). Another correlation observed in our study was a negative association between maternal folic acid intake and maternal milk *Streptococcus* RA. The most likely hypothesis for this correlation is that there is an indirect effect of the folic acid levels consumed by the mother, on the growth of other bacterial species in her milk. Such an effect has been observed previously in the small intestine [56,57]. Williams et al. also reported a negative association between maternal folic acid consumption and milk bacteria concentration; however, their correlation was to a different member of the *Lactobaillales* order, *Lactobacillus*. Of note, *Lactobacillus*, prevalent in their study, was barely detectable in ours, which could explain why we did not detect the latter association. Discrepancies between the two studies may arise from differences in the cohorts, but also from different research methodologies. Dietary data were based on short term food consumption in the Williams study (24 h dietary recall), whereas we used long-term eating patterns (FFQ questionnaires). The Williams study also averaged microbial relative abundances across time before correlation analysis, thereby excluding temporal effects on microbial–dietary interactions.

While there have been recent reports of an association between maternal nutrition during pregnancy and her infant’s gut bacterial composition [58], our study, however, did not find such significant associations at any of the time points, probably due to differences in methodology and the study population.

The negative correlation of *Streptococcus* levels with oleic acid that we observed, together with the positive correlation of *Staphylococcus hominis* levels with medium chain fatty acids, probably results from a higher sensitivity of *Streptococcus* to the antibacterial properties of fatty acids, especially oleic acid, compared to *Staphylococcus*. Indeed, such differences in sensitivity were shown before on closely related species, such as Group A *Streptococcus*, *Staphylococcus aureus* and *S. epidermidis* [54]. While *S. aureus* is considered the predominant etiological agent of lactational mastitis in humans [59], oral *Streptococcus* species are commonly encountered in sub-acute infections [60], and thus maternal nutritional habits could affect the risk of subsequent mastitis.

Previous work that examined the variability of the composition of fatty acids in breast milk from mothers in different countries around the world, and the effect of these differences on the bacterial milk composition, portrayed somewhat different trends [61]. In that study, Kumar et al., reported a strong negative correlation between levels of *Streptococcus* and saturated fatty acids, along with a strong negative correlation, between *Staphylococcus* and monounsaturated fatty acids. It should be noted that the composition of the dominant bacteria in milk microbiomes in that study differed across geographical regions, and therefore the lack of agreement with our results is not surprising. We propose that the extent of the antibacterial effect of fatty acids in the human milk depends on the quantitative relationship between the dominant bacteria in the milk. Another difference that could affect the results is the different form of comparison. While we compared maternal milk bacteria to the concentration of single fatty acids in breast milk, Kumar et al. [61] compared groups of lipids, which may have masked some associations. Country-specific differences aside, our study provides additional evidence that maternal nutrition via its effects on milk lipid content, may affect the composition of the milk microbiome, adding another layer of importance to maternal diet postpartum.

## Figures and Tables

**Table 1:** Interactions between human milk bacteria and food frequency questionnaire (FFQ) nutrition components from the second time point—one-month post-partum.

FFQ Nutrition Components	Bacteria Genus	*p* Value	False Discovery Rate	R
Total polyunsaturated fatty acids	*Streptococcus*	>0.001	>0.001	−0.734
Total monounsaturated fatty acids	*Streptococcus*	0.001	0.098	−0.668
Folic acid	*Streptococcus*	0.001	0.098	−0.669

## Supplementary Materials

The following are available online at https://www.mdpi.com/2072-6643/12/9/2539/s1, Table S1: clinical characteristics of the study participate, Table S2: 21 dietary parameters analyzed from FFQ-normalized by dividing them by the participant’s weight (weight before the pregnancy), Table S3: interactions between human milk bacteria and FFQ nutrition components- one month postpartum, Table S4: interaction-human milk bacteria and FFQ nutrition components-three months postpartum, Table S5: 19 fatty acids detected in the human milk samples, Figure S1: chemical transesterification using acid, Figure S2: comparison between infant gut microbiome and human milk microbiome, Figure S3: inter and intra- subject comparisons between infant’s stool bacterial composition in weighted unifrac distance diagrams.
