# Supplementary material for: Effect of Maternal Diet and Milk Lipid Composition on the Infant Gut and Maternal Milk Microbiomes
Source: Nutrients. 2020 Aug 21;12(9):2539. doi: 10.3390/nu12092539 (PMC7551594; doi:10.3390/nu12092539)
Supplement: Supplementary file 1 [file nutrients-12-02539-s001.pdf]

**Supplemental Table 1:** Clinical characteristics of the study participate												
	**Maternal**							**Infant**				
**Participant number**	**Age (years)**	**BMI (kg/cm^2)**	**Weight gain during pregnancy (kg)**	**Smoking habits**	**Blood type**	**Additive omega 3 consumption**	**Maternal antibiotic treatment**	**Sex**	**Birth weight (kg) (WHO percentile)**	**Abdominal pain**	**Infant antibiotic treatment**	**Siblings**
1	37	27	9	no	A+	no	no	male	3 (23%)	yes	no	yes
2	35	20	17	no	B+	no	no	female	3.4 (64%)	yes	no	yes
3	32	23	15	yes	AB+	no	yes (Synthomycine)	male	3.4 (53%)	no	no	yes
4	25	24	16	no	A+	no	no	female	3.3 (57%)	no	no	yes
5	33	22	13	no	A+	no	no	female	3.1 (42%)	no	no	yes
6	38	23	15	no	B+	no	no	male	3.7 (78%)	yes	no	yes
7	29	20	17	no	A+	yes	yes, during the labor (penicillin)	female	3.2 (51%)	yes	no	no
8	36	20	10	no	O+	no	no	female	2.9 (20%)	yes	no	yes
9	31	21	7	no	O+	no	no	female	3.3 (57%)	yes	no	yes
10	33	20	14	no data	O+	yes	no	female	3.3 (53%)	yes	no	yes
11	35	30	no data	no	A+	no	no	female	3.2 (47%)	yes	no	yes
13	39	21	9	no	A+	yes	no	male	3.4 (54%)	yes	no	yes
14	34	22	8	no	AB+	no	no	female	3.0 (33%)	yes	no	yes
15	30	22	18	no	B+	no	no	female	3.7 (82%)	yes	no	yes
17	27	20	9	no	A+	no	no	male	3.1 (29%)	yes	no	yes
18	32	19	12	no	A+	no	no	female	3.2 (49%)	yes	no	yes
19	29	21	13	no	B+	yes (during 1 month after labor)	no	male	3.4 (58%)	no	no	no
20	34	37	9	no data	B+	no	yes (moxivite forte 3 months after labor)	female	3.2 (44%)	yes	no	yes
21	32	26	no data	no	AB+	no	no	female	2.4 (3%)	yes	no	yes
22	37	19	14	no	O-	no	no	male	3.2 (39%)	yes	no	yes
23	40	32	no data	no data	A+	no data	no data	male	3.8 (83%)	yes	no	yes
24	29	24	15	no	O+	yes	yes, during the labor (penicillin)	female	3.5 (74%)	yes	no	no

Supplemental Figure 1: Chemical transesterification using acid

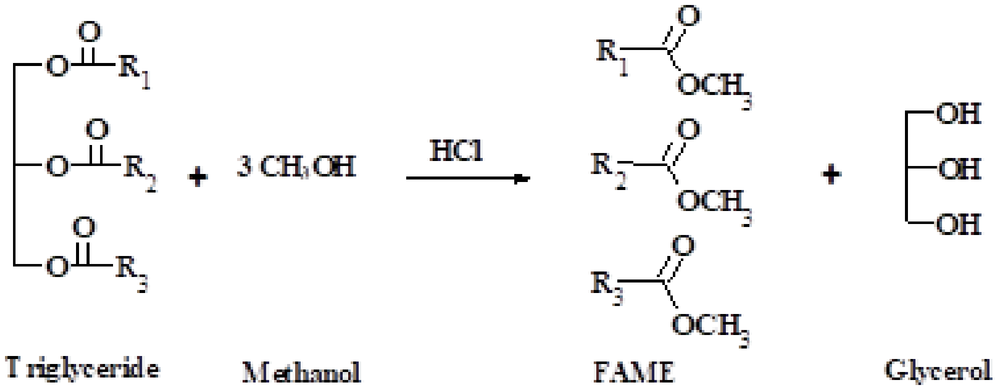

Supplemental Figure 2: Comparison between infant gut microbiome and human milk microbiome

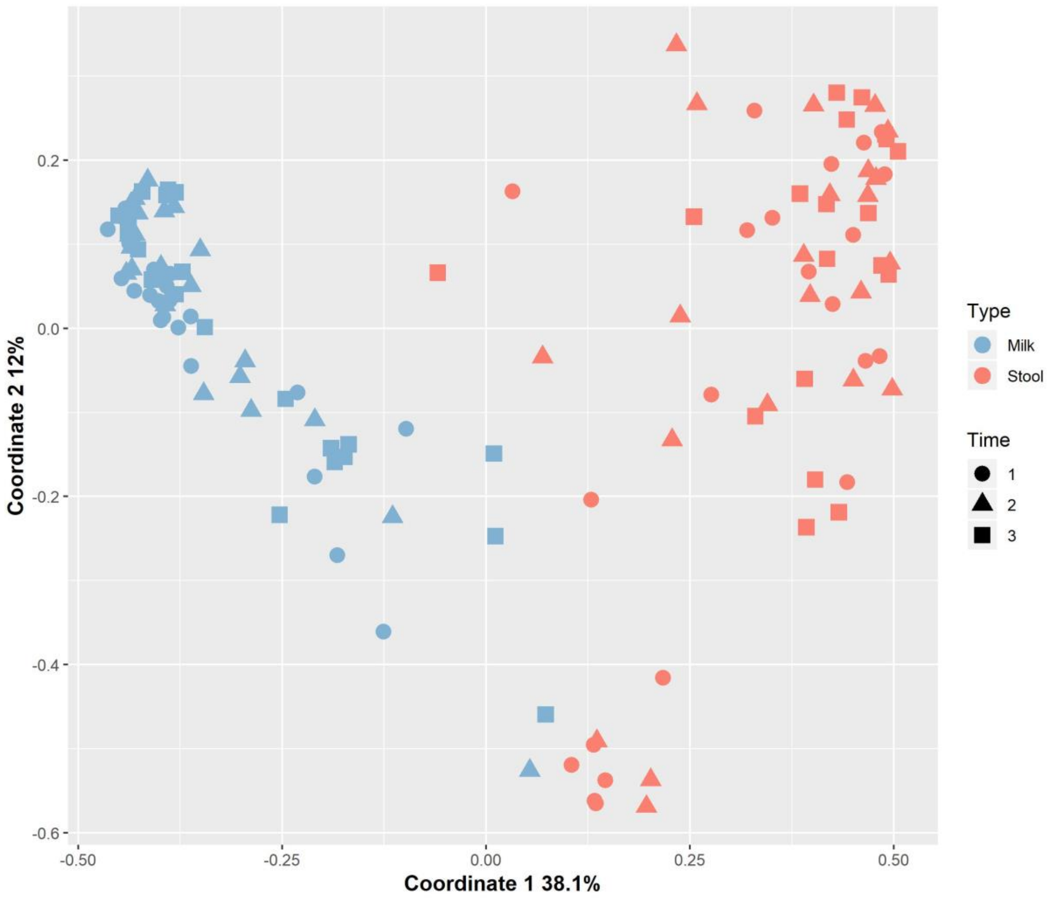

Supplemental Figure 3: inter and intra- subject comparisons between infant's stool bacterial composition in weighted unifrac distance diagrams

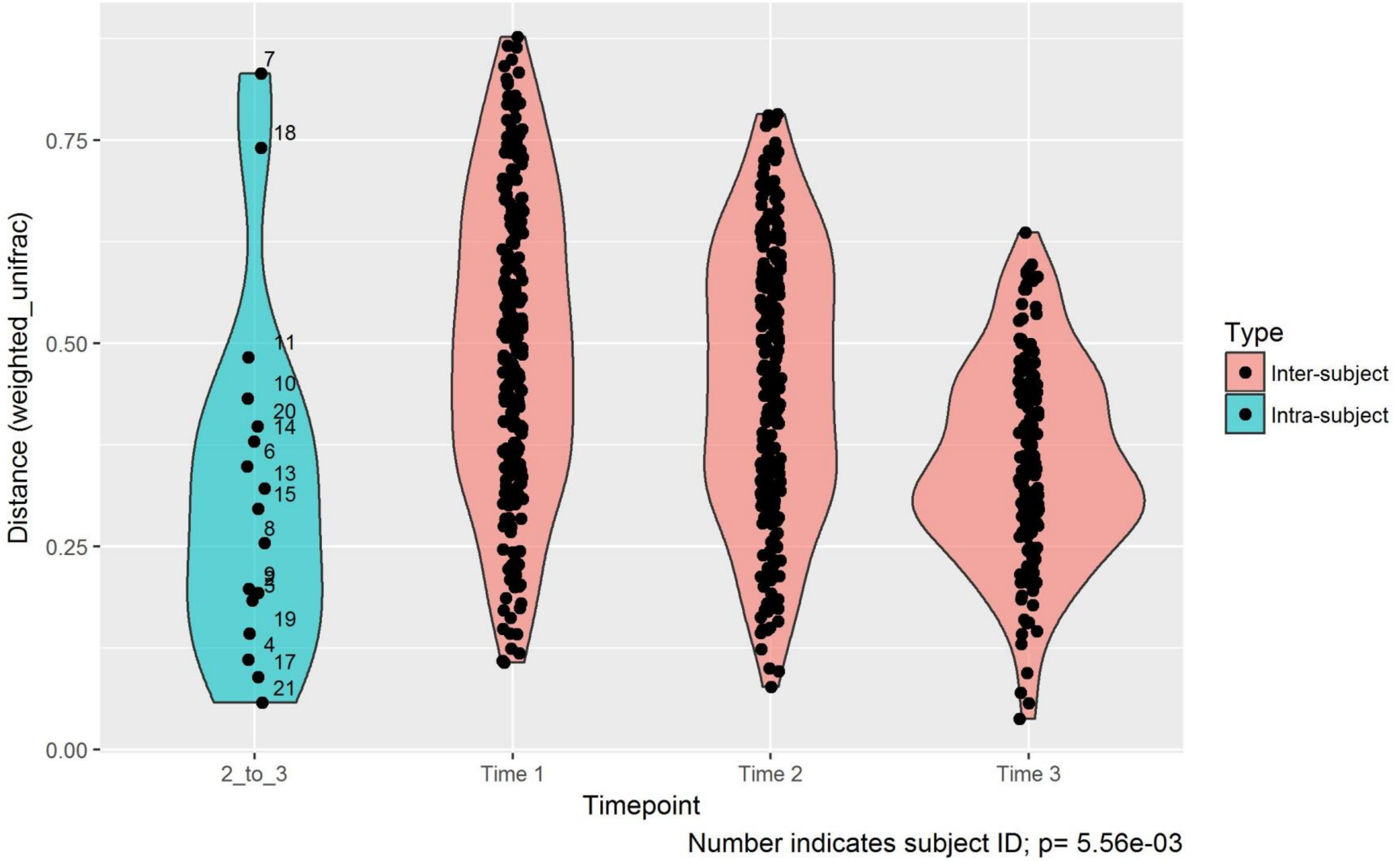

Supplemental Table 2: Interactions between human milk bacteria and FFQ nutrition components*

OTUs' repeats out of all the samples (%)	q	p	rho	ffq	Taxonomic name	OTUs
99%	0	0	-0.698	Fatty_acids_total_monounsaturated	g__Streptococcus; s__Unknown[1]	OTU_30
100%	0	0	-0.706	Fatty_acids_total_polyunsaturated	g__Streptococcus ; s__Unknown[1]	OTU_13
99%	0	0	-0.714	Fatty_acids_total_polyunsaturated	g__Streptococcus; s__Unknown[1]	OTU_33
100%	0	0	-0.721	Fatty_acids_total_polyunsaturated	g__Streptococcus; s__Unknown[1]	OTU_23
99%	0	0	-0.723	Fatty_acids_total_polyunsaturated	g__Streptococcus; s__Unknown[1]	OTU_24
99%	0	0	-0.725	Fatty_acids_total_polyunsaturated	g__Streptococcus; s__Unknown[1]	OTU_15
97%	0	0	-0.726	Fatty_acids_total_polyunsaturated	g__Streptococcus; s__Unknown[1]	OTU_37
99%	0	0	-0.726	Fatty_acids_total_polyunsaturated	g__Streptococcus; s__Unknown[1]	OTU_62
100%	0	0	-0.739	Fatty_acids_total_polyunsaturated	g__Streptococcus; s__Unknown[1]	OTU_1
91%	0	0	-0.746	Fatty_acids_total_polyunsaturated	g__Streptococcus; s__Unknown[1]	OTU_571
99%	0	0	-0.75	Fatty_acids_total_polyunsaturated	g__Streptococcus; s__Unknown[1]	OTU_30
91%	0.034	0.001	-0.65	Fatty_acids_total_polyunsaturated	g__Streptococcus; s__Unknown[1]	OTU_670
100%	0.034	0.001	-0.652	Fatty_acids_total_monounsaturated	g__Streptococcus; s__Unknown[1]	OTU_23
99%	0.034	0.001	-0.653	Folic_acid	g__Streptococcus; s__Unknown[1]	OTU_33
91%	0.034	0.001	-0.656	Fatty_acids_total_polyunsaturated	g__Streptococcus; s__Unknown[1]	OTU_328
90%	0.034	0.001	-0.658	Total_lipid_fat	g__Streptococcus; s__Unknown[1]	OTU_291
99%	0.034	0.001	-0.66	Fatty_acids_total_monounsaturated	g__Streptococcus; s__Unknown[1]	OTU_15
99%	0.034	0.001	-0.667	Total_lipid_fat	g__Streptococcus; s__Unknown[1]	OTU_30
99%	0.034	0.001	-0.671	Fatty_acids_total_monounsaturated	g__Streptococcus; s__Unknown[1]	OTU_24
97%	0.034	0.001	-0.672	Fatty_acids_total_polyunsaturated	g__Streptococcus; s__Unknown[1]	OTU_131
91%	0.034	0.001	-0.675	Fatty_acids_total_monounsaturated	g__Streptococcus; s__Unknown[1]	OTU_571
90%	0.034	0.001	-0.679	Fatty_acids_total_polyunsaturated	g__Streptococcus; s__Unknown[1]	OTU_533
90%	0.034	0.001	-0.685	Fatty_acids_total_polyunsaturated	g__Streptococcus; s__Unknown[1]	OTU_291
100%	0.034	0.001	-0.688	Fatty_acids_total_monounsaturated	g__Streptococcus; s__Unknown[1]	OTU_1
90%	0.034	0.001	-0.689	Fatty_acids_total_monounsaturated	g__Streptococcus; s__Unknown[1]	OTU_291
91%	0.048	0.003	-0.61	Carbohydrate_by_difference	g__Streptococcus; s__Unknown[1]	OTU_328
99%	0.048	0.003	-0.61	Total_lipid_fat	g__Streptococcus; s__Unknown[1]	OTU_62
91%	0.048	0.003	-0.611	Carbohydrate_by_difference	g__Streptococcus; s__Unknown[1]	OTU_670
97%	0.048	0.003	-0.612	Fatty_acids_total_monounsaturated	g__Streptococcus; s__Unknown[1]	OTU_131
100%	0.048	0.003	-0.612	Total_lipid_fat	g__Streptococcus; s__Unknown[1]	OTU_23
91%	0.048	0.003	-0.613	Fatty_acids_total_monounsaturated	g__Streptococcus; s__Unknown[1]	OTU_670
74%	0.048	0.003	-0.613	Folic_acid	f__Gemellaceae; g__; s__Unknown[2]	OTU_132
99%	0.048	0.003	-0.614	Folic_acid	g__Streptococcus; s__Unknown[1]	OTU_30
99%	0.048	0.003	-0.614	Folic_acid	g__Streptococcus; s__Unknown[1]	OTU_62
90%	0.048	0.003	-0.617	Energy	g__Streptococcus; s__Unknown[1]	OTU_291
100%	0.048	0.003	-0.618	Folic_acid	g__Streptococcus; s__Unknown[1]	OTU_1
90%	0.048	0.003	-0.619	Fatty_acids_total_polyunsaturated	g__Streptococcus; s__Unknown[1]	OTU_455
99%	0.048	0.003	-0.621	Carbohydrate_by_difference	g__Streptococcus; s__Unknown[1]	OTU_30
91%	0.048	0.003	-0.622	Fatty_acids_total_polyunsaturated	g__Streptococcus; s__Unknown[1]	OTU_261
99%	0.048	0.003	-0.622	Total_lipid_fat	g__Streptococcus; s__Unknown[1]	OTU_15
99%	0.048	0.003	-0.623	Folic_acid	g__Streptococcus; s__Unknown[1]	OTU_15
91%	0.048	0.002	-0.625	Carbohydrate_by_difference	g__Streptococcus; s__Unknown[1]	OTU_571
99%	0.048	0.002	-0.627	Fatty_acids_total_monounsaturated	g__Streptococcus; s__Unknown[1]	OTU_33
97%	0.048	0.003	-0.627	Fatty_acids_total_monounsaturated	g__Streptococcus; s__Unknown[1]	OTU_37
97%	0.048	0.003	-0.63	Folic_acid	g__Streptococcus; s__Unknown[1]	OTU_37
91%	0.048	0.002	-0.631	Total_lipid_fat	g__Streptococcus; s__Unknown[1]	OTU_571
100%	0.048	0.003	-0.632	Folic_acid	g__Streptococcus; s__Unknown[1]	OTU_13
91%	0.048	0.002	-0.633	Fatty_acids_total_monounsaturated	g__Streptococcus; s__Unknown[1]	OTU_261
100%	0.048	0.002	-0.64	Fatty_acids_total_monounsaturated	g__Streptococcus; s__Unknown[1]	OTU_13
99%	0.048	0.002	-0.642	Fatty_acids_total_monounsaturated	g__Streptococcus; s__Unknown[1]	OTU_62
100%	0.048	0.002	-0.642	Folic_acid	g__Streptococcus; s__Unknown[1]	OTU_23
[illegible]	[illegible]	[illegible]	[illegible]	[illegible]	[illegible]	[illegible]

*Samples from the second-time point - 1 month after delivery

1 99% identity to known sequences in BLASTN database- s_pneumoniae/oralis/mitis/infantis/ oligofermentans

2 99% identity to known sequences in BLASTN database- s_haemolysans/ taiwanensis/parahaemolysans / sanguinis

Supplemental Table 3: Interaction-human milk bacteria and FFQ nutrition components*

OTUs' repeats out of all the samples (%)	Q	p	rho	FFQ variable	Taxonomic name	OTUs
91%	0	0	-0.695	Vitamin_B_12	g__Streptococcus; s__Unknown[1]	OTU_529
73%	0.0922	0.014	-0.525	Vitamin_B_12	g__Rothia; s__mucilaginosa	OTU_17
50%	0.0922	0.014	-0.525	Folic_acid	g__Streptococcus; s__ Unknown[1]	OTU_88
91%	0.0922	0.014	-0.526	Total_lipid_fat	g__Streptococcus; s__ Unknown[1]	OTU_328
100%	0.0922	0.014	-0.526	Fatty_acids_total_saturated	g__Streptococcus; s__ Unknown[1]	OTU_23
97%	0.0922	0.014	-0.526	Fatty_acids_total_saturated	g__Streptococcus; s__ Unknown[1]	OTU_37
91%	0.0922	0.014	-0.527	Folic_acid	g__Streptococcus; s__ Unknown[1]	OTU_261
97%	0.0922	0.014	-0.528	Total_lipid_fat	g__Streptococcus; s__ Unknown[1]	OTU_131
94%	0.0922	0.014	-0.528	Total_lipid_fat	g__Staphylococcus; s__ epidermidis	OTU_150
91%	0.0922	0.014	-0.529	Carbohydrate_by_difference	g__Streptococcus; s__ Unknown[1]	OTU_529
99%	0.0922	0.014	-0.529	Fatty_acids_total_trans	g__Streptococcus; s__ Unknown[1]	OTU_33
90%	0.0922	0.014	-0.529	Fatty_acids_total_saturated	g__Streptococcus; s__ Unknown[1]	OTU_455
94%	0.0922	0.014	-0.53	Fatty_acids_total_saturated	g__Staphylococcus; s__ epidermidis	OTU_150
99%	0.0922	0.013	-0.53	Fatty_acids_total_monounsaturated	g__Streptococcus; s__ Unknown[1]	OTU_62
89%	0.0922	0.013	-0.53	Fatty_acids_total_monounsaturated	g__Staphylococcus; s__ hominis	OTU_69
99%	0.0922	0.014	-0.531	Fatty_acids_total_saturated	g__Streptococcus; s__ Unknown[1]	OTU_30
90%	0.0922	0.013	-0.533	Fatty_acids_total_trans	g__Streptococcus; s__ Unknown[1]	OTU_455
100%	0.0922	0.014	-0.534	Vitamin_B_12	g__Streptococcus; s__ Unknown[1]	OTU_13
97%	0.0922	0.013	-0.534	Fatty_acids_total_trans	g__Streptococcus; s__ Unknown[1]	OTU_131
90%	0.0922	0.013	-0.534	Fatty_acids_total_saturated	g__Streptococcus; s__ Unknown[1]	OTU_291
90%	0.0922	0.012	-0.535	Protein	g__Streptococcus; s__ Unknown[1]	OTU_291
99%	0.0922	0.013	-0.535	Cholesterol	g__Streptococcus; s__ Unknown[1]	OTU_62
90%	0.0922	0.013	-0.535	Fatty_acids_total_trans	g__Streptococcus; s__ Unknown[1]	OTU_533
100%	0.0922	0.012	-0.537	Fatty_acids_total_trans	g__Streptococcus; s__ Unknown[1]	OTU_23
90%	0.0922	0.012	-0.538	Vitamin_B_12	g__Streptococcus; s__ Unknown[1]	OTU_498
90%	0.0922	0.012	-0.539	Total_lipid_fat	g__Streptococcus; s__ Unknown[1]	OTU_455
91%	0.0922	0.012	-0.539	Folic_acid	g__Streptococcus; s__ Unknown[1]	OTU_529
99%	0.0922	0.013	-0.54	Cholesterol	g__Streptococcus; s__ Unknown[1]	OTU_15
100%	0.0922	0.012	-0.543	Protein	g__Streptococcus; s__ Unknown[1]	OTU_1
97%	0.0922	0.011	-0.543	Fatty_acids_total_trans	g__Streptococcus; s__ Unknown[1]	OTU_37
100%	0.0922	0.012	-0.544	Total_lipid_fat	g__Streptococcus; s__ Unknown[1]	OTU_13
91%	0.0922	0.011	-0.544	Energy	g__Streptococcus; s__ Unknown[1]	OTU_529
100%	0.0922	0.012	-0.544	Fatty_acids_total_trans	g__Streptococcus; s__ Unknown[1]	OTU_13
99%	0.0922	0.012	-0.545	Total_lipid_fat	g__Streptococcus; s__ Unknown[1]	OTU_15
94%	0.0922	0.01	-0.546	Protein	g__Staphylococcus; s__ epidermidis	OTU_150
97%	0.0922	0.01	-0.546	Cholesterol	g__Streptococcus; s__ Unknown[1]	OTU_131
90%	0.0922	0.01	-0.547	Fructose	g__Streptococcus; s__ Unknown[1]	OTU_291
91%	0.0922	0.01	-0.548	Protein	g__Streptococcus; s__ Unknown[1]	OTU_261
90%	0.0922	0.01	-0.548	Energy	g__Streptococcus; s__ Unknown[1]	OTU_455

* Samples from the third-time point - 3 months after delivery

1 99% identity to known sequences in BLASTN database- s_pneumoniae/oralis/mitis/infantis/ oligofermentans
